# Reflection-mode acousto-optic imaging using a one-dimensional ultrasound array with electronically scanned focus

**DOI:** 10.1117/1.JBO.25.9.096002

**Published:** 2020-09-03

**Authors:** Lukasz J. Nowak, Wiendelt Steenbergen

**Affiliations:** University of Twente, Faculty of Science and Technology, Biomedical Photonic Imaging Group, Enschede, The Netherlands

**Keywords:** acousto-optic imaging, reflection mode, ultrasound linear array, electronic focus scanning

## Abstract

**Significance:** Practical implementation of acousto-optic imaging (AOI) encounters difficulties that prevent it from rapid adoption in clinical use. In many practical medical applications, the region of interest may be accessed only from one side, and using a water tank for coupling is not feasible. The solution might be to use reflection-mode imaging with an electronically scanned ultrasound (US) focus. Such an approach, however, entails considerable challenges.

**Aim:** The possibilities of detecting and localizing light-absorbing inclusions inside turbid media by combining reflection-mode AOI conducted using a one-dimensional US array with electronic scanning of the US focus are investigated experimentally and signal processing algorithms that could be used for this purpose are introduced.

**Approach:** We determine the speckle contrast decrease due to the acousto-optic effect as a function of the US focal point coordinates. Different signal postprocessing techniques are investigated.

**Results:** A significant decrease in the determined speckle contrast difference values is observed due to the presence of light-absorbing inclusions. However, local minima occur in the plots only under specific conditions. Subtracting individual distributions and determining symmetry deviations allow for localizing the inclusions.

**Conclusions:** Detection and localization of optically distinct regions are possible using the introduced approach. Signal postprocessing is required in a general case.

## 1 Introduction

Pathological changes in tissues, such as tumors, can often be detected by their distinctive optical properties. This fact is exploited by various medical imaging techniques for fast and noninvasive diagnostics and therapy guidance. Unfavorably, if such pathological changes are localized deeper than a few millimeters inside the body, their detection using purely optical means becomes challenging due to the high scattering of light in tissues.^1^ Ultrasound (US) imaging can be used to visualize deeper anatomical structures; however, it is only capable of detecting boundaries of regions with high enough acoustic impedance contrast. Also, it does not provide any information regarding the diagnostically relevant optical properties of the region of interest.

Acousto-optic imaging (AOI) is a technique that can potentially overcome the limitations of purely optical and acoustical medical imaging modalities by utilizing the effects of the interaction between focused US and light scattered in a tissue. Acoustic wave propagating through a medium causes periodic displacement of optical scatterers and changes in the resulting interference patterns of the detected light. A theoretical description of the underlying physical phenomena including various possible mechanisms of interaction was presented by Leutz and Maret^2^ and Wang.^3^ For optimal performance, the light source should have the coherence length close to the mean optical path length, i.e., of the order of at least several centimeters for tissue-like media.^4^ Numerous studies on AOI have been published since the early 1990s. Recently, several comprehensive reviews^1^^,^^5^^,^^6^ have been published.

The majority of the studies in the field concern transmission-mode AOI. In such a configuration, a light source and a detector are located on both sides of the investigated sample, while the US transducer is placed perpendicular to the transmitter–detector axis. This allows for mapping the determined values proportional to the intensity of detected modulated light into one-dimensional (1D) plots or two-dimensional (2D) images in which the presence of light-absorbing inclusions manifests itself as local minima. Using this kind of setup, Lai et al.^7^ demonstrated the possibility of imaging through 9.4 cm tissue-like phantom. Bocoum et al.^8^ and Laudereau et al.^9^ used a 1D US array to modulate the light with focused and plane wave US pulses. Using different image reconstruction algorithms, they were able to obtain reconstructed fluence rate maps of the detected light with locations of 2- and 3-mm light-absorbing inclusions clearly visible as local minima on the plots. Hussain et al.^10^ used for AOI six fiber bundles evenly distributed around a sample to transmit and receive light pulses. The US pulses were transmitted using a custom-made concave transducer array. The introduced technique allowed for determining the local fluence rate distribution inside agar gel phantoms, *ex-vivo* tissue samples, and freshly euthanized mice and for using the obtained data to improve the quality of photoacoustic images.

Unfavorably, in many practical medical applications, the region of interest may be accessed from one side only. Thus, the light source, detector, and US transducer would have to be located next to each other. Theoretical aspects of such an approach were studied by Granot et al.^11^ The light fluence rate inside tissue or other highly scattering media will have a banana-shaped distribution between the optodes for illumination and detection. The corresponding analytical solutions can be found for relatively simple cases of infinite or semi-infinite, homogeneous media and parallel optode positions.^12^

One of the solutions for visualizing light-absorbing regions using a reflection-mode AOI setup might be to keep the relative positions of the optical transmitter, receiver, and US transducer in fixed locations, while physically moving the investigated sample. If no electronic focus scanning is applied, then mutual distributions of the acoustic pressure field and light fluence rate remain constant for given medium parameters. When an absorbing inclusion is introduced within the insonified and illuminated volume, a decrease in the intensity of modulated light is detected. The changes in the measured signal with the changes of relative sample positions can thus be directly associated with variances in optical properties at given coordinates. This was done by Lev and Sfez,^13^^–^^15^ who used two fibers arranged in reflectance geometry for AOI of different phantoms and tissues. The light was modulated using continuous-wave (CW) US with a fixed focus, positioned perpendicular to the optical transmitter–receiver plane. The scanning process was performed by physically moving the samples relative to the fibers and US transducer. They have demonstrated that detection of light-absorbing inclusions inside the investigated samples is possible using this approach. Hisaka and Sasakura,^16^ Kim et al.,^17^ and Hong-Bo et al.^18^ presented reflection-mode AOI setups with a light source and detector located above the US transducer, all at fixed positions on the same side of the sample. The US focus was also fixed, and the scanning was performed by mechanically moving the sample inside a water tank^17^^,^^18^ (the Hisaka and Sasakura study^16^ does not provide details on coupling to the sample). The studies demonstrated that it is possible to detect light-absorbing inclusions inside phantoms and tissue samples using such a measurement configuration.

The aim of this study is to investigate experimentally possibilities of detecting and localizing light-absorbing inclusions inside turbid media by combining reflection-mode AOI conducted using a 1D US array with electronic scanning of the US focus and to introduce signal processing algorithms that could be used for this purpose. Some of our initial results on this topic were previously published as conference proceedings.^19^ In contrast to the other studies on reflection-mode AOI, the imaging is performed with the US transducer and optodes in direct contact with the sample, on the same side, and without physical movement. The motivation for such an approach stems directly from limitations imposed by many potential, real-life medical applications (access to a sample from one side only, impossibility of using a water tank for coupling) and its possible benefits in terms of scanning time (determined by signal-to-noise ratio and required number of illumination events and not by the probe translation time). Electronic scanning of focus introduces specific challenges, related to the differences in acoustic pressure field distribution with variable US focal point coordinates on the background of constant light fluence rate distribution (as the position of optodes does not change). Due to the banana-shaped fluence rate distribution of the detected light, the presence of optically distinct inclusions can manifest itself in quite an unpredictable manner, depending on the size and location of the inclusion. As will be shown further, local minima on the fluence rate maps reconstructed using the introduced approach occur only under specific conditions, which are investigated and discussed. We also introduce various signal postprocessing techniques, such as investigations of symmetry deviations, to detect and localize inclusions in more general cases. To better exploit and demonstrate the benefits of combining acoustics and optics, we developed acoustically homogeneous, opaque phantoms with optically distinct inclusions. We demonstrate that it is possible to detect and obtain cues on localization of those inclusions using the described setup.

## 2 Materials and Methods

**Fig. 1:**
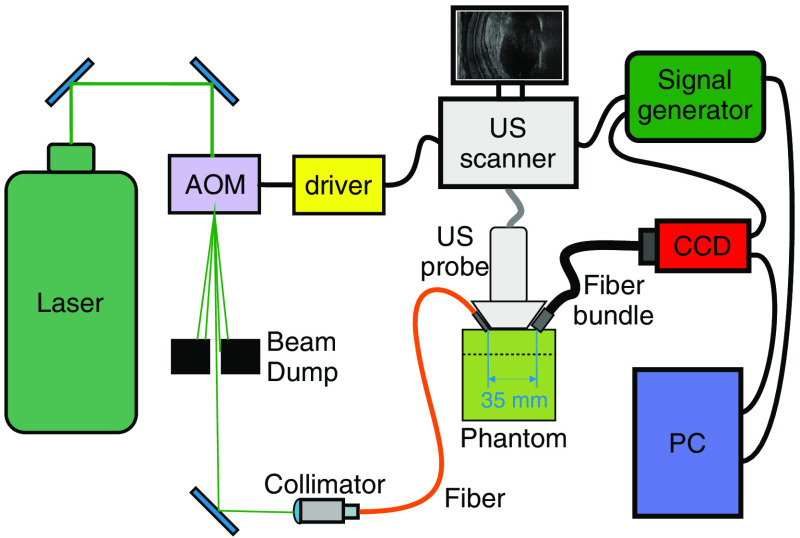
The experimental setup. AOM, acousto-optic modulator; CCD, charge-coupled device camera.

The experimental setup used for measurements is shown in Fig. 1. A 532-nm CW laser with 6.5-W output power (Coherent Verdi 6) was used as a light source. An acousto-optic modulator (AOM) (Gooch & Housego, R23080-3-LTD) was used to convert a CW beam into 1-μs pulses triggered by an US scanner (Verasonics Vantage 256) through a gated 80-MHz output signal driver (NEOS Technologies, 21080-2DM). The laser pulses were coupled into a 200-μm multimode fiber connected to an integrated acousto-optic probe.

**Fig. 2:**
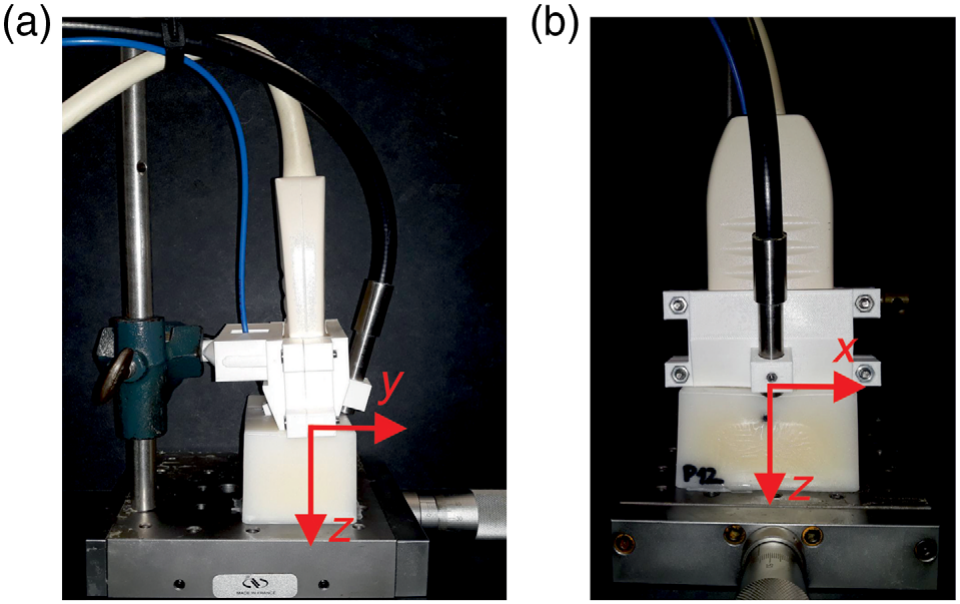
The integrated probe used for the experimental investigations on top of a phantom: (a) side view and (b) front view. The arrows indicate the orientation of the adopted coordinate system (associated with the probe). The phantom dimensions (x×y×z) are $70\times 48\times 35\;{\mathrm{mm}}^{3}$.

The integrated probe used in the experimental investigations is shown in Fig. 2. The probe comprised a transmitting fiber, a 128 element US linear array (ATL L7-4), and a circular fiber bundle with a diameter of 4 mm for collecting light. All of the components were held together by a custom-made, 3D-printed enclosure. The fibers were at an angle of 20 deg relative to the probe, inclined toward each other, 35 mm away, and oriented centrally and perpendicular to the US transducer array. The integrated probe was pressed directly against the surfaces of the investigated samples. The adopted coordinate system, also shown in Fig. 2, is associated with the probe. It is assumed that the middle point of the US array has coordinates (x,y,z)=(0,0,0) and that the transducer elements are arranged along the x axis. The positive values of the z coordinates correspond to the sampling depth.

The fiber bundle exit facet was positioned ∼35  mm in front of the camera charge-coupled device (CCD) array (Allied Vision Stingray F-125B), on which a speckle pattern is formed. The camera was triggered by an external signal generator with a frequency of 10 Hz. The same triggering signal was fed to the US scanner, which further triggers the laser pulses. The laser pulses were delayed relative to the US pulses by a time period determined independently for every single focal point in such a way that the acoustic wave reached its focus in the middle of the 1-μs illumination period (in other words, if the first US pulses of a sequence were fired at t=0 and for a given focal point coordinate, they would reach the focus at $t=t_{f}$, then the laser pulse was triggered at $t=t_{f}-\frac{1}{2}t_{i}$, where $t_{i}$ is the illumination time). The camera was connected to a computer for data acquisition.

For every captured frame, we determined the speckle contrast value C, defined as^20^

$$C=\frac{\sigma }{\left\langle I\right\rangle },$$ (1)

where σ stands for standard deviation of the pixel values and ⟨I⟩ is their mean value. An acoustic wave propagating inside an irradiated, diffusive medium interacts with the light by inducing vibrations of the optical scatterers and refractive index changes. The resulting light modulation will lead to a decrease in the observed speckle contrast. This decrease is proportional to the acoustic power.^3^^,^^20^ We determined the speckle contrast difference (SCD) values between pairs of subsequent frames: one without US signal present during illumination and the following one with the focused acoustic wave.

The signal-to-noise ratio is in general very low as the fraction of the modulated and detected light is also low. Thus, the studies in the field usually employ averaging over several tens or even thousands of frames. We captured 100 frame pairs for every imaging point. The exposure time was 50 ms, during which 50 laser pulses (accompanied with the synchronized US pulses for every second frame) were transmitted at 1-ms intervals.

Changing the US focus of the linear array was performed by applying various delays to the individual transducer elements (determined by the US scanner software based on the focal point coordinates provided within the control scripts). All 128 elements were used, which allowed for obtaining ∼2-MPa peak pressure at a focal point (value determined by hydrophone measurements in a water tank). Each US pulse consisted of 2 cycles with the center frequency of 5.208 MHz. The total insonified volume is determined by propagation time, applied delays, pulse duration and location, and parameters of sources. Within this volume, a complex interference pattern can be observed with distinct global maximum at the focus.

The experimental investigations on AOI were carried out on cuboid phantoms with dimensions of $70\times 48\times 35\;{\mathrm{mm}}^{3}$. The phantoms were cast from polyvinyl chloride plastisol (PVCP, Lure Flex firm by Lure Factors) with an addition of 3.5  mg/ml of titanium dioxide (Aldrich, particle size 44  μm) for optical scattering. The acoustic and optical properties of PVCP can be suited to approximate the properties of different human tissues.^21^^,^^22^ Our goal was to develop acoustically homogeneous, opaque phantoms with optically distinct inclusions. The phantoms were cast in two-step processes. First, hot PVCP (i.e., heated above its transition temperature, ∼180°C) was poured into a cubical mold with a cylindrical metal bar inside. The bar was stretched centrally between opposite walls of the mold and removed from the phantom after curing. Next, the same PVCP material with the addition of black ink (Lure Factors, std. black) was heated and injected into the remaining hole inside the PVCP block. Phantoms without inclusions were also cast as a reference for measurements. The inclusions inside phantoms were neither visible on US images nor from the surface. The propagation velocity of acoustic waves inside phantoms was determined to be 1700  m/s. The extent of the US focus inside the phantoms in the axial (z) direction was thus ∼0,65  mm, while the pulse propagation distance within the laser illumination period was 1.7 mm. Optical properties of the PVCP were determined based on spectrophotometer measurements (Shimadzu UV-2600) using the inverse adding-doubling method.^23^ The optical scattering coefficient $\mu_{s}$ was determined to be $∼45\;{\mathrm{cm}}^{-1}$. The absorption coefficient $\mu_{a}$ was found to be negligible for PVCP without ink and equal to $∼17\;{\mathrm{cm}}^{-1}$ for the same material with ink. Four different phantoms were cast: one without inclusion; one with a 4-mm-diameter inclusion oriented centrally along the shorter (35 mm) edge and located 10 mm below the top surface; and two with 2- and 3-mm-diameter inclusions oriented centrally along the longer (70 mm) edge and located 5.5 and 5 mm below the top surface, respectively. Different geometries were developed based on the analysis of results of subsequent measurements to test various hypotheses regarding imaging capabilities of the system—as will be explained in detail in Sec. 3. The use of the solid phantoms allowed for investigating the underlying phenomena in a controlled manner and minimizing the limitations imposed by the speckle decorrelation time, which would be much more strict in the case of tissue samples.

## 3 Results

**Fig. 3:**
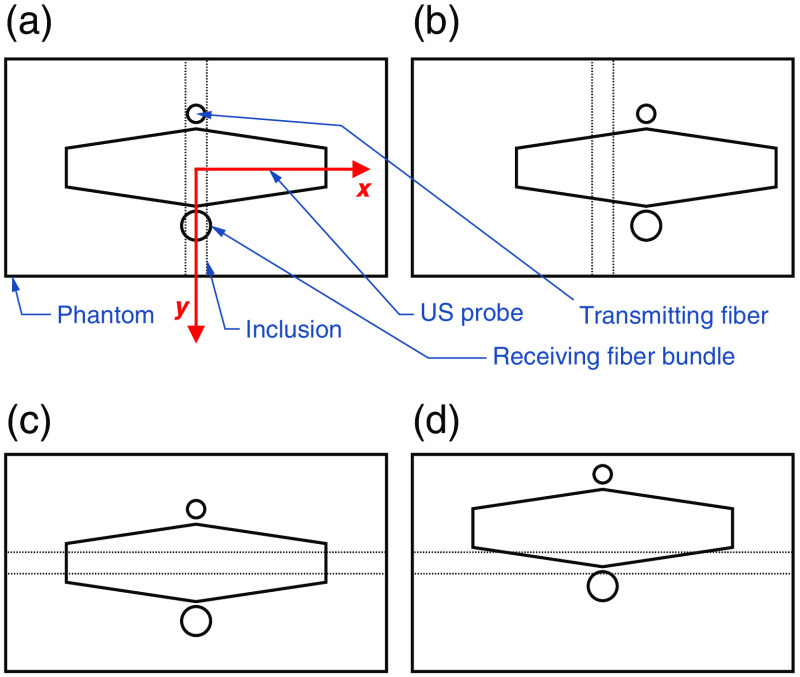
Schematic drawing of different relative probe–phantom positions (top view).

SCD values were determined for different phantoms and various probe positions relative to the inclusions. Some of the considered geometries are shown in Fig. 3. The speckle contrast value in the absence of the US varied between different cases and was in the range of ∼0.31 to 0.42. It was limited by the noise in the adopted system configuration as a consequence of the trade-off between the intensity of the detected light, its fluence rate distribution (extent of the sensitivity region of the imaging system), and the required camera exposure time. The highest base speckle contrast values were observed for the phantoms and probe locations for which light absorption was also highest due to the low mean intensity values in the denominator in Eq. (1). The results were stable and repeatable in all cases.

**Fig. 4:**
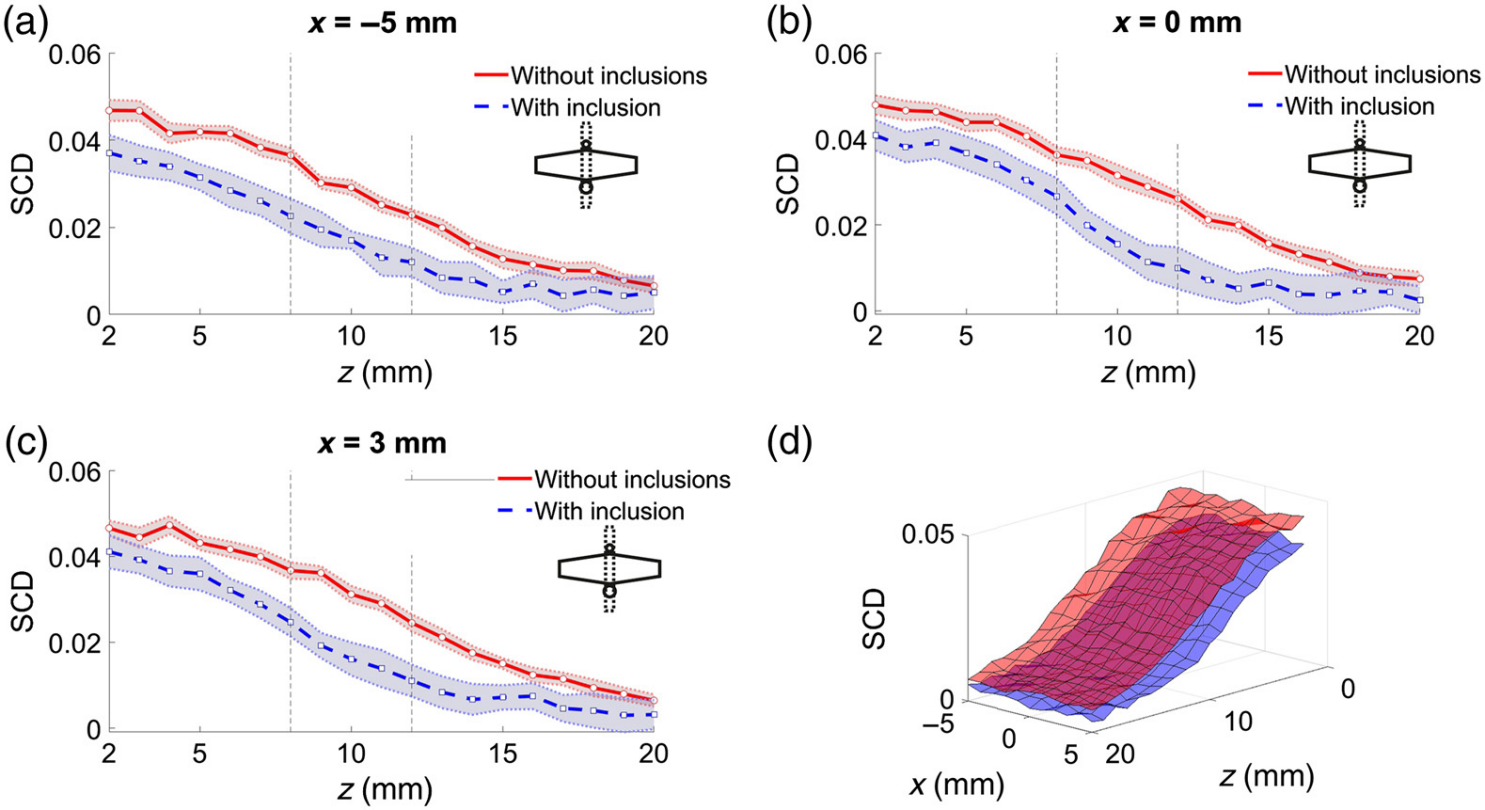
SCD values as functions of US focal point coordinates x and z for phantoms without any inclusion and with a 4-mm-diameter inclusion at z=10  mm. (a)–(c) Shaded areas indicate standard deviation, and vertical lines indicate location of the inclusion. (d) Top surface corresponds to the phantom without any inclusion.

Figure 4 shows SCD values as functions of US focal point coordinates obtained for two different phantoms: one without any inclusion, and the other one with a 4-mm-diameter inclusion 10 mm below the surface (center of the cylinder), oriented during the measurements along the y axis [i.e., perpendicular to the US transducer array and along the optical transmitter–detector axis, as shown in Fig. 3(a)]. The probe was positioned at the center point, above the inclusion. Figures 4(a)–4(c) present scans along the z axis with different US focal point x coordinates. Surface plot (d) presents the results of 2D scans in the xz plane. The top surface in Fig. 4(d) corresponds to the phantom without any inclusion.

**Fig. 5:**
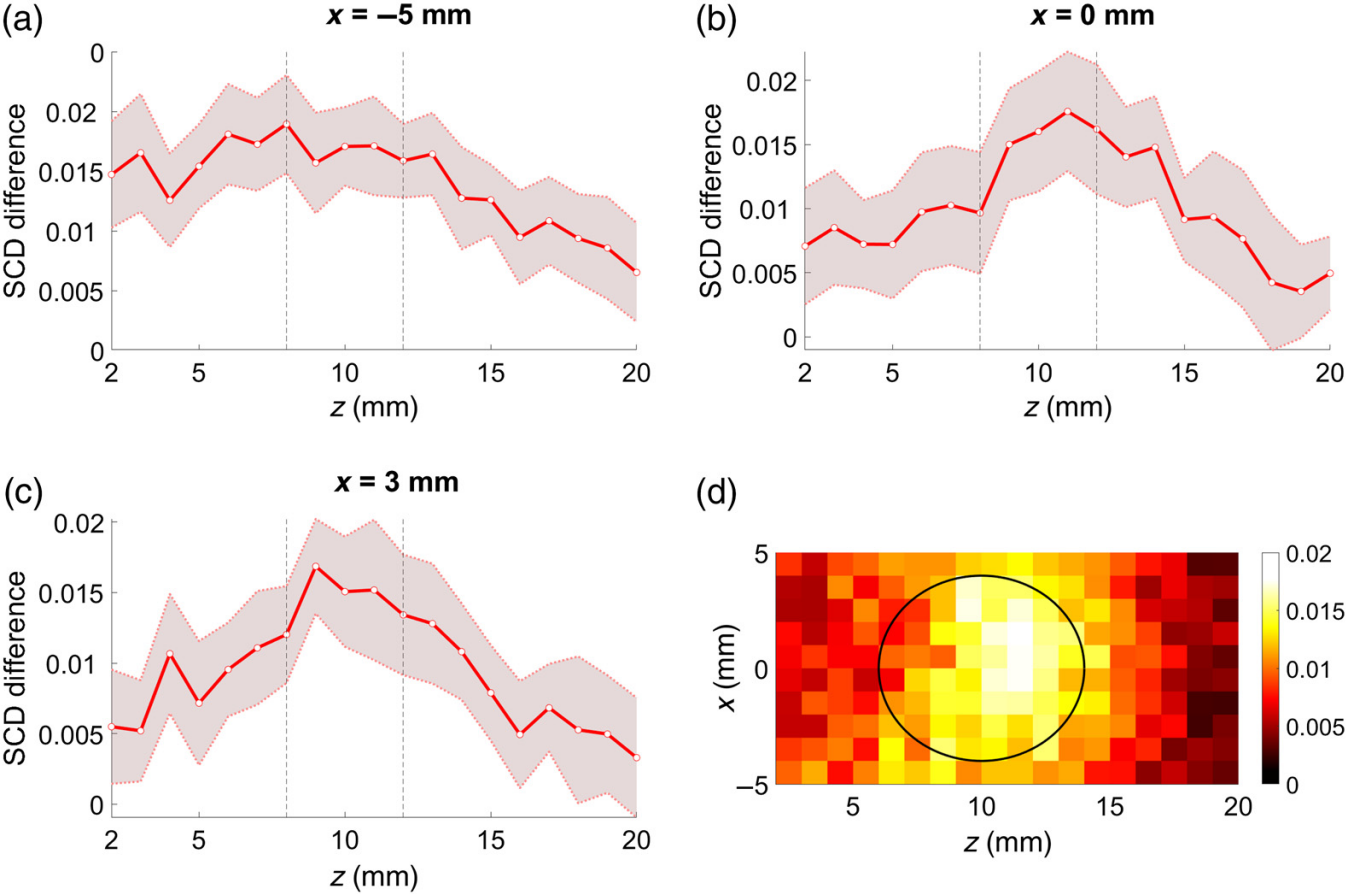
Results of subtraction of the SCD values obtained for phantoms without any inclusion and with a 4-mm-diameter inclusion at z=10  mm, presented as functions of US focus coordinates: line scans along z direction (a)–(c) and cross section in xz plane (d). Shaded areas indicate standard deviation, and black outlines indicate position of the inclusion.

As shown in Fig. 4, the SCD values obtained for the phantom with the inclusion are in every case lower compared with the corresponding results obtained for the homogeneous phantom. The distinction between the phantoms is clearly possible. The shapes of the plots are also slightly different; however, visual analysis of the results does not allow for conclusions about the location of the optically absorbing region as there are no distinct local minima. This information can be further extracted from the considered datasets by subtracting the results obtained for both phantoms. Figures 5(a)–5(d) present the differences between the corresponding SCD values from Fig. 4. In this case, the local maxima are clearly visible for US focal point coordinates within the location of the inclusion.

**Fig. 6:**
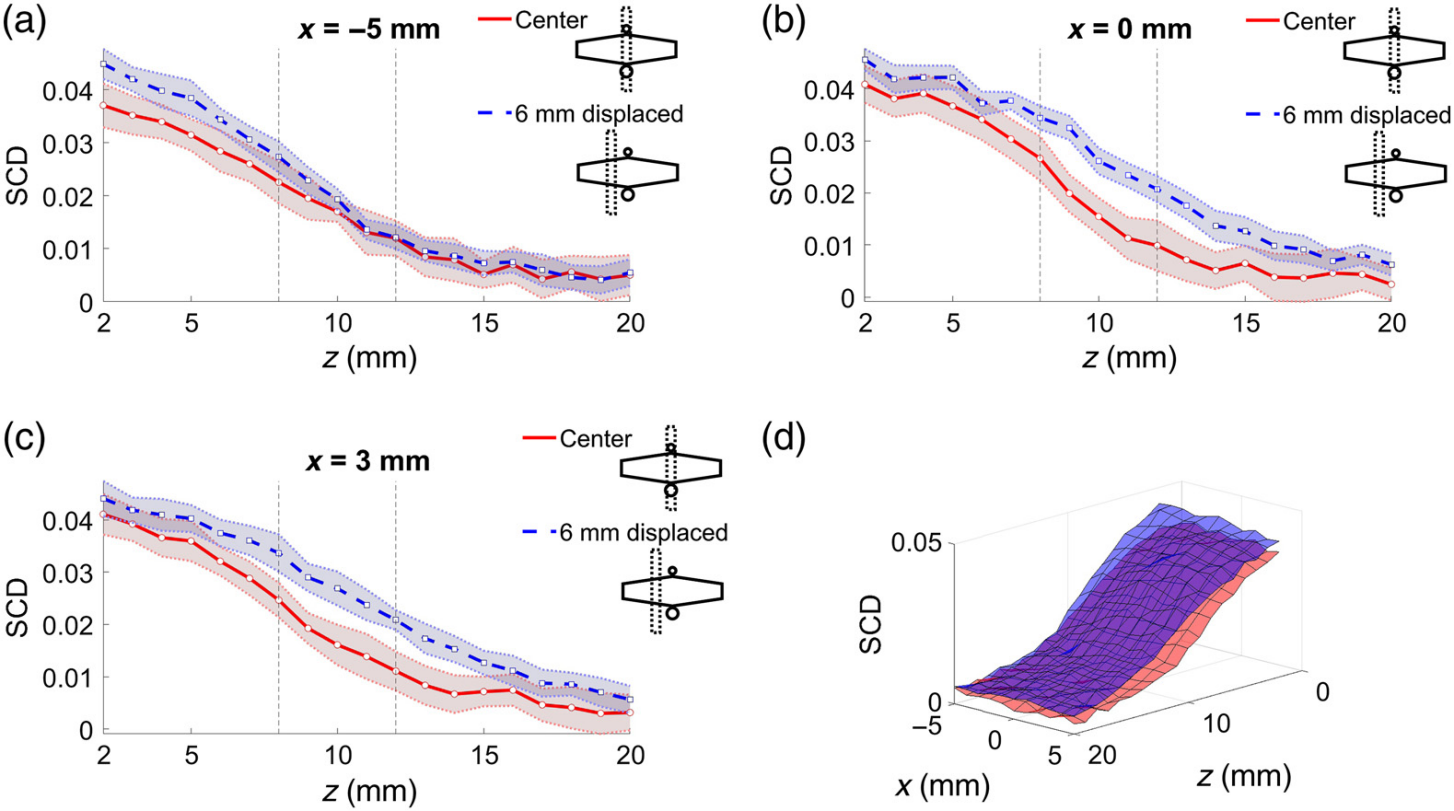
SCD values as functions of US focal point coordinates x and z determined for a phantom with a 4-mm-diameter inclusion at z=10  mm and two probe positions: directly above the inclusion and 6 mm displaced. (a)–(c) Shaded areas indicate standard deviation, and vertical lines indicate location of the inclusion. (d) Top surface corresponds to the probe position 6 mm displaced.

Figure 6 shows the comparison of SCD values obtained for the same phantom with a 4-mm-diameter inclusion 10 mm below the surface, oriented along the y axis, for two different probe positions: directly above the inclusion and displaced 6 mm along the x axis, as shown in Figs. 3(a) and 3(b), respectively. Thus, in the latter case, the inclusion was parallel and 6 mm off the optical transmitter–detector axis. Figures 6(a)–6(c) show scans along the z axis with different US focal point x coordinates. Figure 6(d) shows the results of 2D scans in the xy plane. The top surface corresponds to the case of the probe displaced 6 mm relative to the inclusion. As it can be seen, moving the probe aside results in an increase of the obtained SCD values within the whole considered range of the US focus coordinates.

**Fig. 7:**
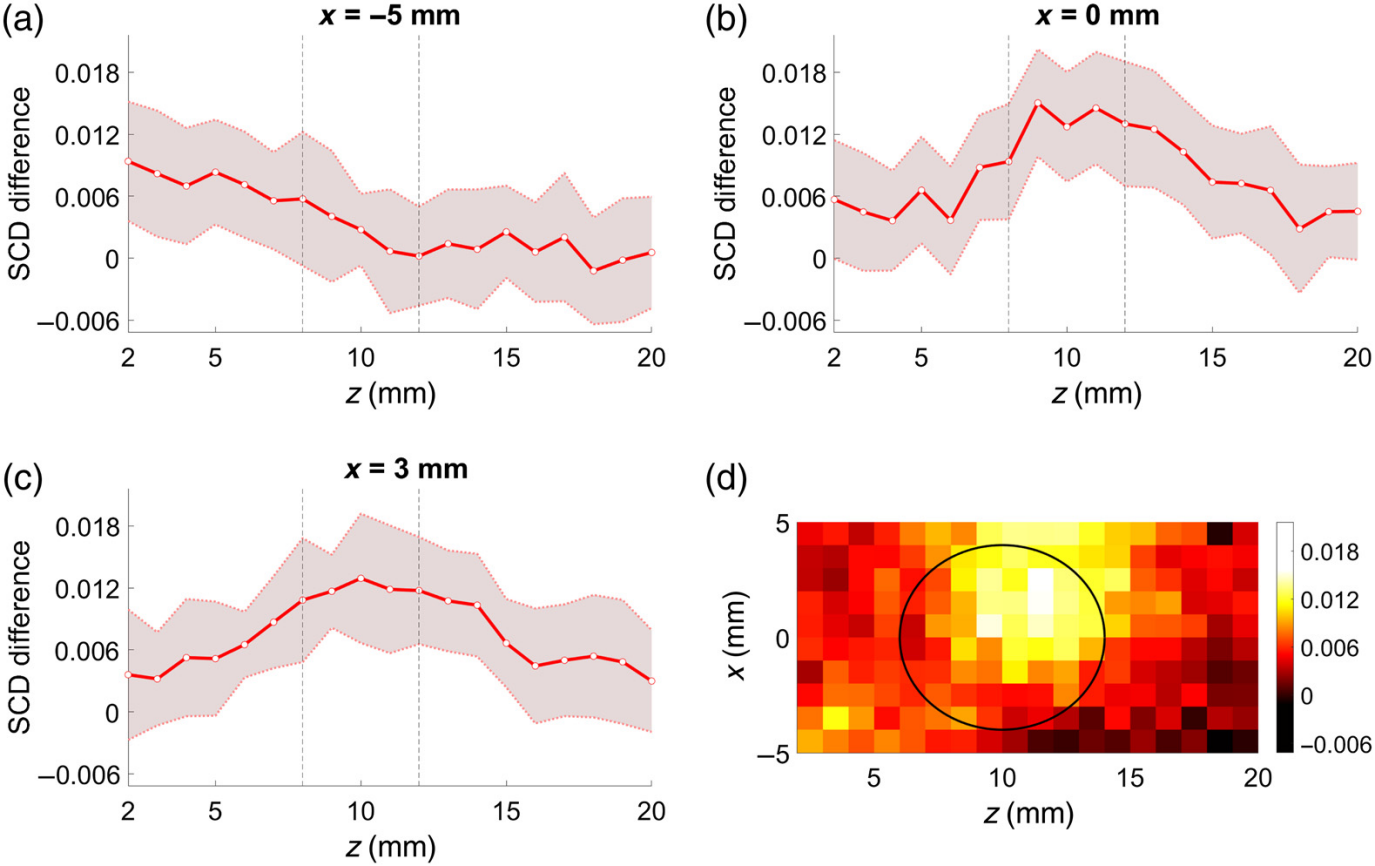
Results of subtraction of the SCD values obtained for a phantom with a 4-mm-diameter inclusion at z=10  mm and two probe positions: directly above the inclusion and 6 mm displaced, presented as functions of US focus coordinates: line scans along z direction (a)–(c) and cross section in xz plane (d). Shaded areas indicate standard deviation, and black outlines indicate position of the inclusion.

Figures 7(a)–7(d) show the differences between the corresponding SCD values from Fig. 6. Again, such an analysis reveals distinct maxima for US focal point coordinates within the location of the inclusion.

The results shown in Figs. 4 and 6 allow for a clear distinction between the considered phantoms and relative probe-inclusion positions based on the obtained SCD values. Determining the presence of an optically absorbing region and obtaining cues on its location within the sample is only possible by comparing different datasets. This in turn involves physical displacement of the probe. To fully exploit the benefits of electronic scanning of the US focus, extracting such information directly from a single measurement is desired. To address this issue, we investigated the symmetry deviations of the SCD distribution on the xz plane in the case in which an inclusion was positioned off the center axis of the probe (x=0, y=0). We define an asymmetry coefficient ${\mathrm{AC}}_{\chi }$ as

$${\mathrm{AC}}_{\chi }(z)=\mathrm{SCD}(\chi ,z)-\mathrm{SCD}(-\chi ,z),$$ (2)

where SCD(χ,z) and SCD(−χ,z) denote the speckle contrast difference values determined for US focal points coordinates (x=χ,z) and (x=−χ,z), respectively.

**Fig. 8:**
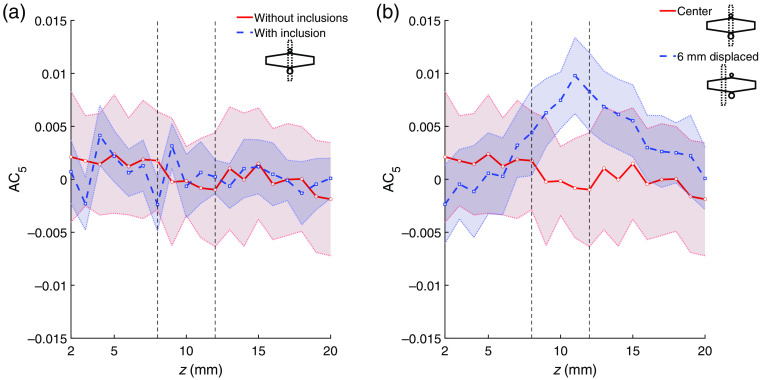
Asymmetry coefficient ${\mathrm{AC}}_{5}$ as a function of imaging depth, determined for phantoms without any inclusion and with a 4-mm-diameter inclusion at z=10  mm, oriented along the y axis and located centrally below the probe (a) and for the same phantom with an inclusion for two different probe positions (b): at the center point of a phantom and 6 mm displaced. Vertical lines indicate location of the inclusion.

Figure 8 shows examples of ${\mathrm{AC}}_{5}(z)$ values determined for phantoms without any inclusion and with a 4-mm-diameter inclusion 10 mm below the surface, oriented along the y axis and located centrally below the probe [Fig. 8(a)], and for the same phantom with an inclusion for two different probe positions [Fig. 8(b)]: at the center point of a phantom, and 6 mm displaced [as shown in Figs. 3(a) and 3(b)]. The determined values fluctuate around zero for all cases except the one in which the inclusion is present and located off the optical axis of the probe. The US focusing depth for which the maximum of the ${\mathrm{AC}}_{5}(z)$ plot is observed corresponds to the actual depth of the absorbing region within the sample.

**Fig. 9:**
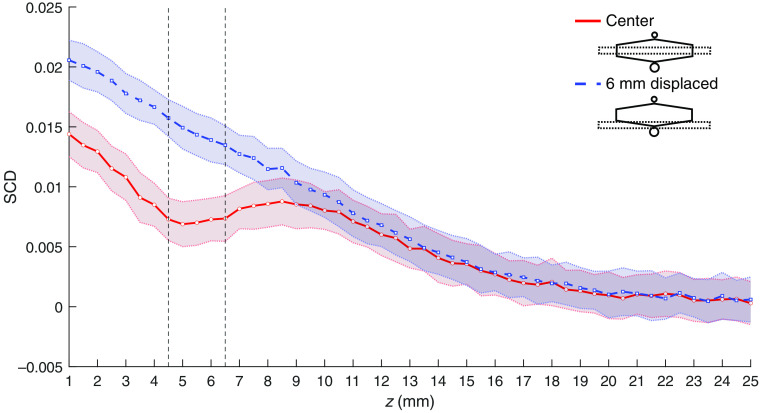
SCD values obtained for a phantom with a 2-mm-diameter inclusion at z=5.5  mm, oriented along the x axis and for two probe positions: directly above the inclusion and 6 mm displaced. Shaded areas indicate standard deviation, and vertical lines indicate location of the inclusion.

**Fig. 10:**
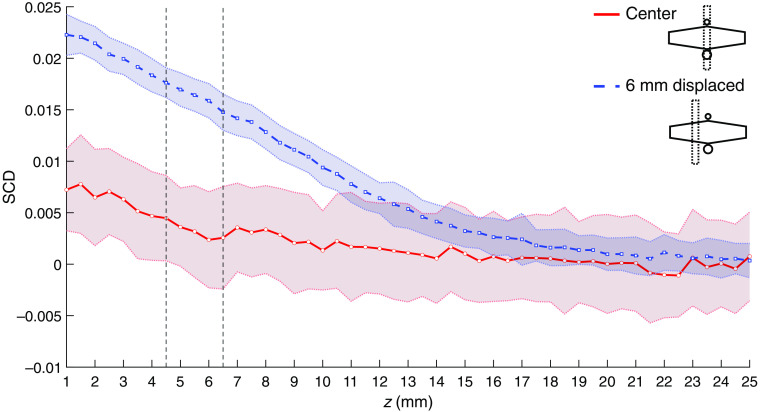
SCD values obtained for a phantom with a 2-mm-diameter inclusion at z=5.5  mm, oriented along the y axis and for two probe positions: directly above the inclusion and 6 mm displaced. Shaded areas indicate standard deviation, and vertical lines indicate location of the inclusion.

We also hypothesized that it should be possible to observe the local minima of the SCD values in some specific cases of sufficiently small absorbing inclusions localized within regions of relatively high illumination. To test this hypothesis, we performed measurements on a phantom with a 2-mm-diameter inclusion 5.5 mm below the surface, oriented along the x axis, i.e., in parallel to the US array and perpendicular to the optical transmitter–detector axis [as in Fig. 3(c)]. The orientation of inclusion was chosen this way to minimize the total amount of the absorbed light. The SCD values obtained for probe positions at the center and displaced 6 mm in the y direction [as shown in Figs. 3(c) and 3(d)] are shown in Fig. 9. When the probe is directly above the inclusion, a distinct local minimum in the SCD is clearly visible. The analogous results obtained for the case in which the phantom is rotated by 90 deg, i.e., when inclusion is parallel to the optical transmitter–detector axis [similar as in Figs. 3(a) and 3(b)], are shown in Fig. 10. In this case, the dip is not visible anymore.

**Fig. 11:**
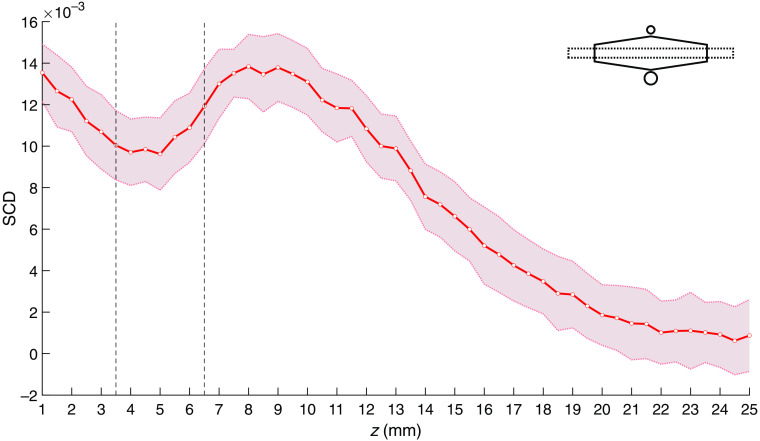
SCD values obtained for a phantom with a 3-mm-diameter inclusion at z=5  mm, oriented along the x axis. Shaded areas indicate standard deviation, and vertical lines indicate location of the inclusion.

The local minimum of the determined SCD values is even more distinct in the case of an inclusion slightly larger in diameter (3 mm) and located 0.5-mm shallower (i.e., at the depth of 5 mm), as shown in Fig. 11.

**Fig. 12:**
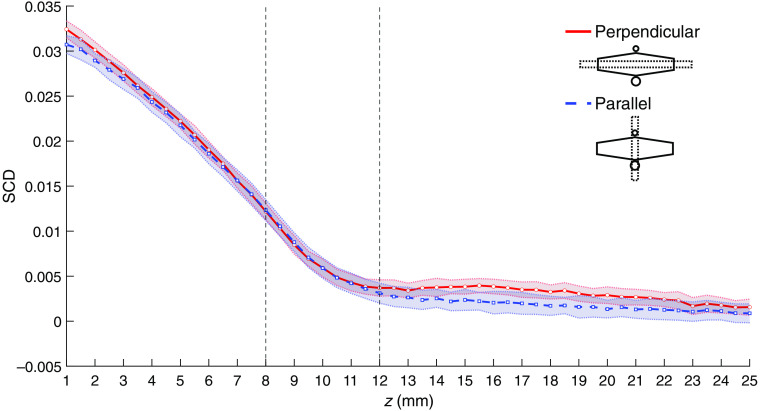
SCD values obtained for a phantom with a 4-mm-diameter inclusion at z=10  mm for two different orientations: parallel and perpendicular to the optical transmitter–detector axis. Shaded areas indicate standard deviation, and vertical lines indicate location of the inclusion.

Figure 12 shows the SCD values as functions of the US focal point z coordinate for the phantom with a 4-mm-diameter inclusion located 10 mm below the surface and two different orientations: when the inclusion is parallel to the optical transmitter–detector axis [as in Fig. 3(a)], and for the phantom rotated by 90 deg, when the inclusion is perpendicular to the optical axis [similar as in Fig. 3(c)]. A weak local minimum can be observed in the latter case at the focusing depth close to the lower boundary of the inclusion.

## 4 Discussion

This study hypothesizes that it is possible to detect and localize light-absorbing inclusions inside acoustically homogeneous, optically opaque samples using a reflection-mode AOI system with a 1D linear US array and electronic scanning. To test this claim, we developed various phantoms fulfilling the assumed conditions, and we determined the SCD values for different US focal point coordinates. The hypothesis holds in light of the presented results of measurements. In all of the investigated cases, the presence of an inclusion in a region below the probe manifests itself by a significant SCD drop, compared with a case with no inclusion or when it is present away from the optical axis of the probe. The character of those changes depends on the size of the absorbing region and its absorption coefficient relative to the one of the surrounding medium, as well as on its location and orientation relative to the probe.

Comparison of the results obtained for phantoms without any inclusion and with a 4-mm-diameter inclusion located directly under the probe, 10 mm below the surface, and oriented along the optical axis allows for an immediate and clear distinction between these two cases. As shown in Fig. 4, the drop in the determined SCD values is clearly visible within the whole range of the considered US focus depth values. If only one set of data corresponding to the phantom with the inclusion is analyzed, the presence and location of the absorbing region would be difficult to determine. The SCD curves shown in Fig. 4 do not have any significant local minima that could act as an adequate indicator. A reference is thus needed. Results of the subtraction of SCD values obtained for the considered phantoms, shown in Fig. 5, give a clear indication of the actual location of the optical inhomogeneity inside the analyzed sample.

Similar results can be obtained using only one phantom and two different probe positions, as shown in Figs. 6 and 7. Again, a drop in the SCD values can be observed within the whole range of US focal point coordinates if the inclusion is located directly below the center of the probe, compared with the case with the probe 6 mm displaced [i.e., as in Figs. 3(a) and 3(b)]. Results of the subtraction of the SCD values obtained for the corresponding US focal point coordinates and different probe locations give a clear indication on the presence and location of the inclusion, as shown in Fig. 7. In this case, we utilize the observation that the influence of inclusion is weaker when it is positioned off the optical axis of the probe, and thus we can use such a measurement as a reference point in a similar manner as the phantom without any inclusion. Considering the potential practical implementation of the imaging system, using one sample and two probe positions within the range of several millimeters instead of two different samples is advantageous.

It was stated at the beginning that avoiding physical movement of the probe or sample during measurements is desired. The results shown in Figs. 5–7 required such a movement; however, they were obtained using only two probe positions for the whole scan. The imaging was performed by electronically scanning the US focus, with all of the time-wise benefits of such an approach. Also, no water tank was required, which should be considered advantageous in terms of potential applications.

We also investigated the symmetry deviations of the determined SCD values in the case in which an inclusion is positioned off the optical transmitter–detector axis. We introduced the asymmetry coefficient ${\mathrm{AC}}_{\chi }$, defined with Eq. (2). The results of measurements indicate that analysis of ${\mathrm{AC}}_{\chi }$ values can provide important cues not only for detection but also for localization of optically absorbing regions within an investigated sample. This has been illustrated in the example of ${\mathrm{AC}}_{5}$ plots as functions of the US focusing depth, shown in Fig. 8. In the case of a phantom without any inclusions or with an inclusion located directly below the probe, the determined ${\mathrm{AC}}_{5}$ values fluctuate around zero. If the probe is displaced and the investigated sample does not reveal symmetry about the optical axis anymore, then a significant local extremum can be observed. The type of the extremum (in this case, it is maximum) indicates if the inclusion is to the left or to the right of the center of the probe. The location of the maximum corresponds to the actual depth of the inclusion. The described information is, in this case, extracted from a single scan, i.e., without the need to physically move the probe or a sample.

The presence of local minima in the SCD values may be used for localization of optically absorbing regions. In our experiments, a local minimum was observed only in cases of some phantoms and probe orientations, which leads to the conclusion that it occurs only under specific conditions. The SCD values determined for different US focal point coordinates are proportional to the product of the fluence rate of the detected light and acoustic pressure amplitude squared,^20^^,^^24^ integrated over the whole illuminated volume. Assuming that the considered phantoms are acoustically homogeneous, the acoustic pressure field distribution for specific US focus coordinates was identical for all considered cases. Thus, the observed differences between the SCD plots result only from the differences in fluence rate distributions of the detected light. The local minimum in the SCD values can be observed if a region within an illuminated volume is characterized with a significantly larger optical absorption coefficient than the surrounding medium and if its size and spatial orientation relative to the probe allow for forming a local maximum of the fluence rate of the detected light in its vicinity. In the analyzed cases, such a situation occurs for 2- and 3-mm-diameter inclusions, located 5 and 5.5 mm beneath the surface and oriented perpendicular to the optical axis of the probe (Figs. 9 and 11). The described condition is no longer met if the orientation of the inclusion overlaps with the optical axis of the probe (Fig. 10) or if the size and depth of the inclusion are too large (Fig. 12).

Using a reflection-mode AOI, it is possible not only to obtain information on the fluence rate distribution of the detected light but also to map the local total fluence rate variations within an analyzed sample. This can be achieved using collocated optodes, as described in Refs. 10 and 25. However, such an approach would be very challenging using the system introduced in this study. In this study, the optodes are in direct contact with a highly scattering sample. Thus, the maximum of the fluence rate distribution would occur at close proximity to the transmitting and receiving fibers, limiting significantly the extent of the sensitivity region of the imaging system. Positioning optodes at the opposite sides of the US probe shifts the maximum of the fluence rate distribution of the detected light to the US focusing plane, increasing system performance.

The required scanning time using the described system with electronically scanned US focus is the product of the number of imaging points and the number of frame pairs captured per point, divided by the camera frame rate. In the described cases, it ranged between 2 min (for a 40 point line scan) and 20 min (400 points cross-section plane scanning). The imaging process may be accelerated using more powerful, shorter laser pulses (or tandem nanosecond pulses, such as described in Ref. 26) and decreasing the camera exposure time while increasing the frame rate. Higher intensity of the detected light could also allow for decreasing the number of frames for averaging, while maintaining the signal-to-noise ratio. It should also be noted that the illumination time should be lower than a decorrelation time of a medium to avoid blurring of the recorded interference patterns and thus decreasing of the measured speckle contrast.

## 5 Summary

We have demonstrated that, using the described reflection-mode AOI system with a 1D US array, it is possible to detect optically distinct inclusions inside acoustically homogeneous, opaque phantoms and to obtain cues on their localization. The presented integrated AOI probe is used in direct contact with investigated samples. It requires neither a water tank for coupling nor access from multiple sides. An electronically scanned US focus provides the potential for fast imaging within 2D cross-section planes.

Under favorable conditions (geometry of the optically absorbing region within the sample and its orientation relative to the probe), it is possible to detect and localize an inclusion based on a single AOI scan. This can be done either from the presence of a local minimum on the determined SCD values distribution or by investigating deviations in the symmetry of these distributions. We have introduced an asymmetry coefficient defined as the difference between the SCD values calculated for US focal points located symmetrically about the center axis of the probe and demonstrated that it could be a useful quantity for analyzing AOI data. In all considered cases, the local extrema indicating positions of optically distinct regions were observed between the actual boundaries of an inclusion, within approximately ±1  mm around its center.

However, in a general case, such information could not always be extracted from the obtained results. For instance, if the inclusion is too large or located too deep or directly below the center of the probe, the described features might not be distinctive enough to determine its presence. In such cases, as we have also demonstrated in our study, the SCD difference between two different AOI scans might still provide a clear indication on the location of the optically absorbing region.
